# Trends in upper gastrointestinal diagnosis over four decades in Lusaka, Zambia: a retrospective analysis of endoscopic findings

**DOI:** 10.1186/s12876-015-0353-8

**Published:** 2015-10-06

**Authors:** Violet Kayamba, Edford Sinkala, Stayner Mwanamakondo, Rose Soko, Boniface Kawimbe, Beatrice Amadi, Isaac Zulu, Jean-Baptiste Nzaisenga, Themba Banda, Chipasha Mumbwe, Evans Phiri, Philip Munkonge, Paul Kelly

**Affiliations:** TROPGAN, Department of Medicine, University of Zambia School of Medicine, University Teaching Hospital, Nationalist Road, Lusaka, Zambia; Copperbelt University School of Medicine, Ndola, Zambia; Department of Surgery, University Teaching Hospital, Lusaka, Zambia; Blizard Institute, Barts & The London School of Medicine, Queen Mary University of London, 4 Newark Street, London, E1 2AD UK

**Keywords:** Endoscopy, Africa, Non-communicable disease, HIV, Gastroduodenal disorders, Peptic ulceration, Cancer

## Abstract

**Background and aims:**

There a shortage of robust information about profiles of gastrointestinal disease in sub-Saharan Africa. The endoscopy unit of the University Teaching Hospital in Lusaka has been running without interruption since 1977 and this 38-year record is largely intact. We report an analysis of endoscopic findings over this period.

**Methods:**

Written endoscopy records from 29th September 1977 to 16th December 2014 were recovered, computerised, coded by two experienced endoscopists and analysed. Temporal trends were analysed using tables, graphs, and unconditional logistic regression, with age, sex of patient, decade, and endoscopist as independent variables to adjust for inter-observer variation.

**Results:**

Sixteen thousand nine hundred fifty-three records were identified and analysed. Diagnosis of gastric ulcer rose by 22 %, and that of duodenal ulcer fell by 14 % per decade. Endoscopically diagnosed oesophageal cancer increased by 32 % per decade, but gastric cancer rose only in patients under 60 years of age (21 % per decade). Oesophageal varices were the commonest finding in patients presenting with haematemesis, increasing by 14 % per decade in that patient group. Two HIV-related diagnoses, oesophageal candidiasis and Kaposi’s sarcoma, rose from almost zero to very high levels in the 1990s but fell substantially after 2005 when anti-retroviral therapy became widely available.

**Conclusions:**

This useful dataset suggests that there are important trends in some endoscopic findings over four decades. These trends are not explained by inter-observer variation. Reasons for the divergent trends in incidence of peptic ulceration and apparent trends in diagnosis of upper gastrointestinal cancers merit further exploration.

## Background

A longitudinal perspective on disease trends is not only fascinating, but also essential to understanding aetiology and pathogenesis. The epidemiology of upper gastrointestinal disease in industrialised populations is evolving rapidly [1–3]. Falling rates of peptic ulceration [4] and gastric cancer [2] are attributed to declining prevalence of *Helicobacter pylori* infection [5, 6]. Oesophageal squamous cell carcinoma remains the most predominant worldwide, although in developed countries there is a shift to adenocarcinoma [3, 7–9]. Data from Asia are similar to those in Western countries with a gradual decrease in peptic ulceration and *H. pylori* infection [10–13]. There is also a reported reduction in peptic ulceration in Latin America [14].

In Africa, the shortage of data impedes understanding of the current epidemiology of upper gastrointestinal disorders [15] and there is almost no insight into trends over time. Towards the end of the twentieth century, the concept of the ‘African enigma’ gained currency, signifying that a high prevalence of *H. pylori* infection was not matched by a high burden of attributable upper gastrointestinal disease [16]. There is abundant evidence that *H. pylori* infection is common throughout Africa [17–19]. However, we and others have found that the burden of upper gastrointestinal disorders is actually considerably higher than it appeared at first, and a community survey in Lusaka using endoscopy found that peptic ulcers were present in 4 % of a poor urban community, only half of which were symptomatic [18]. Cancer registry data across Africa are incomplete [20], but such data as exist indicate that oesophageal carcinoma incidence is high [21]. Record linkage methods, used effectively in industrialised countries, are feasible in Africa but the lack of computerised medical records precludes optimal analysis of existing data [22]. As primary health care in sub-Saharan Africa is patchy, and frequently inaccessible to all but the urban middle class, there are few data on the burden of communicable and non-communicable gastrointestinal disorders. Endoscopy units, which are an important source of data on upper gastrointestinal disease [23], are much less well developed in Africa than in industrialised countries, and not many of them have records going back for more than a few years.

The University Teaching Hospital in Lusaka (UTH) has served the population of this capital city, and indeed the whole country and beyond, since the 1970s. The endoscopy unit in UTH has grown from small beginnings in 1977 to a unit which now delivers a daily service and offers diagnostic and therapeutic upper and lower gastrointestinal endoscopy. One of the authors (SM) was nurse leader of the unit from 1985 to 2012 and has preserved an almost-complete set of written records of diagnostic procedures, a record which began with the founding of the unit in 1977. In the belief that this probably unique archive may provide insight into emerging trends of disease in this large African city, we have analysed all the available records from the unit. The records include the periods before the emergence of the Human Immunodeficiency Virus (HIV) infection and during the epidemic before and after the availability of antiretroviral therapy.

## Methods

### Endoscopy records

We retrieved all available endoscopy reports since the inception of the unit from 1977 to 2014. Reports for all endoscopy procedures from 1977 to 2009 were written by hand on proformas, and have been kept together. From 2009, records were entered on computer, but paper copies of all entries are printed off and kept with the older written records to avoid loss of data in the event of computer breakdown. The hand-written records comprise a wide range of free text entries, in contrast to which the computerised entries are largely pre-coded. Written records were missing for the whole year 1986, and for the period April-December 2008; endoscopies were performed over these periods, but the records could not be found. Records were entered into Excel spread sheets by five people, with verbatim entry of free text for all clinical fields. No names were entered. Identification of endoscopist signatures was verified by one of the nurses (SM) who had been in charge of the unit from 1985 to 2012. The free text in all written records was then transferred into Stata 13 (Stata Corp, College Station, TX) and coded by two experienced endoscopists (PK and VK). The University of Zambia Biomedical Research Ethics Committee granted exemption from ethics review for publication of this retrospective analysis on 22nd January, 2015; consent was not obtained for this from patients in view of the 40-year time span, and this was considered when the waiver was obtained.

### Coding

Free text entries were reviewed and coded into categories of: (i) endoscopist; (ii) indication for endoscopy; (iii) findings in the oesophagus; (iv) findings in the stomach; and (v) findings in the duodenum. All endoscopists were coded, but only those who performed more than 500 procedures are shown and the rest grouped together as ‘guest doctor’. Upper gastrointestinal haemorrhage was coded by searching the “Indications” text entries for words including “haematemesis”, “bleeding”, “melaena”, “vomiting blood”, “suspected bleeding varices”, or “suspected Mallory-Weiss”. Anaemia was coded using search terms including “anaemia”, “pallor” and all spelling variants; dysphagia was coded by searching for “dysphagia”, “difficulty swallowing” and similar terms, and abdominal pain using terms such as “pain”, “abdo. pain”, “suspected PUD” and suchlike. All findings in oesophagus, stomach or duodenum were coded into categories. Specific search terms for oesophageal candidiasis included “candida”, “candidiasis”, “thrush” or “white plaques”. Varices in oesophagus or stomach were searched by “varices” with or without any other text. For oesophageal, gastric or duodenal ulcers, terms included “ulcer”, “ulceration”, “DU”, “GU”. The term “erosions” was coded separately as it was frequently associated with “inflammation” which (depending on site) was coded with oesophagitis, gastritis or duodenitis. For oesophageal, gastric or duodenal cancer, the terms included “tumour”, “growth”, “lesion” and “malignant”, but “polyp”, “nodule”, and “smooth growth” were coded as polyp/benign lesion. Kaposi’s sarcoma (KS), which has a distinctive appearance was coded separately and the terms such as “KS”, “Kaposi’s” “Kaposi’s sarcoma” were used. It was not possible to confirm the diagnoses of cancer or KS by histology, as these records were not available for most of the time period under analysis. Children were defined as 16 years of age or less.

### Data analysis

Time was analysed in ‘decades’, which although not perfect 10 year intervals (Table 1) were felt to be useful intervals for analysis. Diagnostic categories were treated as categorical variables and analysed by frequency, as percentage of records available, and by logistic regression with sex, age less than 45 or 60 years, endoscopist and decade as independent variables. Significance testing was carried out using the *χ*^2^ test or Fisher’s exact test as appropriate and *P* < 0.05 considered significant.

**Table 1:** Patient characteristics and indication for endoscopy, by decade

	1977–1985^a^	1987–1996	1997–2006	2007–2014	
n	1674	2627	3388	9264	
Male: female	998:569	1606–945	1771–1462	4445:3977	<0.0001
Female %	36	37	45	47	
Age (median, IQR)	33, 26–46	35, 27–45	35, 27–47	39, 29–54	0.001
Age groups in years:					
Child (under 16)	13 (1)	31 (1)	66 (3)	311 (4)	
16–44	888 (72)	1828 (74)	1697 (68)	4646 (57)	
45–59	215 (17)	434 (17)	464 (19)	1697 (21)	
60 and over	121 (10)	192 (8)	277 (11)	1453 (18)	<0.0001
Indication:					
Pain	1192 (71)	1957 (75)	1.912 (56)	4566 (49)	<0.0001
Dysphagia	64 (4)	99 (4)	222 (8)	961 (11)	<0.0001
Haematemesis	193 (12)	194 (8)	276 (22)	1062 (12)	<0.0001
Anaemia	30 (2)	63 (3)	14 (1)	187 (2)	<0.0001

Percentages are shown in brackets

^a^No data from 1986 retrieved, and the last decade is only 8 years so the decades are therefore not uniform

## Results

A total of 16,953 records were found, entered and verified, spanning the 38-year period 1977–2014. Among those 15,773 had the patients’ sex recorded and 8820 (56 %) were men and 6593 (44 %) women (Table 1). Median age was 37 years (interquartile range 28–50 years) with no difference between the sexes. There were 16,725 records in which an oesophageal finding was recorded and interpretable, 16,100 with a gastric finding, and 14,704 with a duodenal finding. The great majority of procedures were carried out without sedation, which is a policy decision as resource constraints limit the number of staff to supervise recovery safely. We are aware of two deaths in the procedure room over this period, one unexplained and one in a severely anaemic patient being scoped for gastrointestinal (GI) haemorrhage; there were no deaths in children though anecdotally we are aware of several instances when benzodiazepine sedation had to be reversed with flumazenil.

### Endoscopists and inter-observer variation

**Fig. 1:**
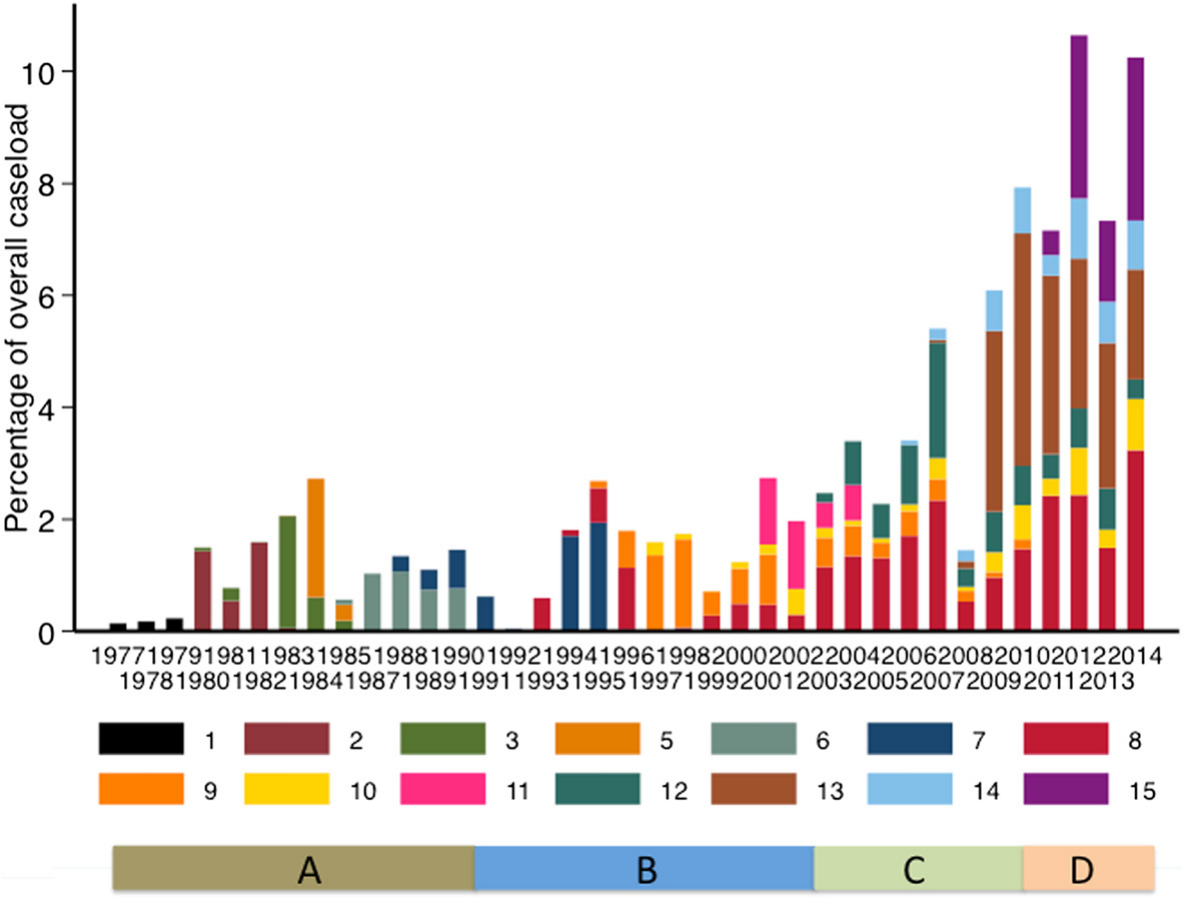
Contributions of individual endoscopists over the period 1977–2014. Only those endoscopists who contributed over 500 procedures are shown, except where they were the only doctors performing endoscopy at that time. The other 23 doctors are not shown, for clarity, but contributed 2656 records to the total. The bars at the bottom indicate the equipment in use over the whole period, beginning with non-immersible endoscopes (**a**) from 1977 to 1991, then immersible fibre-optic Olympus endoscopes (**b**) from 1991 to 2003, then fibre-optic Pentax endoscopes with camera viewing system (**c**) from 2003 to 2010, and lastly Pentax high-definition video endscopes (**d**) from 2010 to date

A total of 37 doctors performed most of the endoscopies (31 physicians, 5 surgeons and 1 paediatrician) and their records could be easily recognised. There were a few others included simply as ‘guest doctor’. Thirteen of the 37 doctors contributed over 500 procedures each, and five contributed over one thousand each. Many of these span several years, with one contributing over two decades (Fig. 1). With respect to inter-observer variation there is clear evidence that doctors differed with respect to diagnosis (Table 2). There was also variation by medical specialty. Surgical endoscopists were more likely to diagnose oesophageal cancer (3.9 % vs 3.2 % by physicians; *P* = 0.048), and less likely to diagnose duodenal ulcer (7.1 % vs 9.2 %; *P* < 0.0001), oesophageal varices (3.3 % vs 5.4 % respectively; *P* = 0.0001) or normal findings (34 % vs 66 %; *P* = <0.0001). The single paediatrician, who carries out procedures on both adults and children, was nevertheless less likely to diagnose cancer (doctor 10 in Table 2). However, the frequent diagnosis of oesophageal candidiasis by doctors 9 and 11, and lack of such by doctors 1, 2, 3 and 5, actually reflects the period over which they worked (Fig. 1), as HIV-related disorders were virtually unknown before about 1984.

**Table 2:** Principal endoscopists over time and diagnostic variation

Doctor	Period	n	Children	Candida	Varices	Reflux	OCA	GU	GCA	DU	KS	Normal
1	1977–1980	78	0	0	1 (1)	6 (8)	0	8 (10)	0	12 (16)	0	9 (12)
2	1980–1983	520	9	0	25 (5)	10 (2)	12 (2)	35 (7)	15 (3)	87 (16)	0	63 (13)
3	1980–1985	445	6	6 (1)	14 (3)	2	13 (3)	13 (3)	6 (1)	47 (11)	2	215 (52)
5	1983–1985	347	5	3 (1)	22 (6)	0	15 (4)	44 (13)	5 (1)	15 (4)	0	68 (28)
6	1985–1990	532	7	1 (0.2)	31 (6)	7 (1)	17 (3)	23 (5)	12 (2)	77 (15)	1	183 (38)
7	1988–1998	819	18	51 (6)	22 (3)	86 (11)	10 (1)	29 (4)	8 (1)	62 (8)	3	256 (33)
8	1993–2014	3499	101	202 (6)	220 (7)	63 (2)	115 (3)	210 (6)	110 (3)	398 (12)	24	1502 (50)
9	1995–2010	1199	14	123 (10)	34 (3)	21 (2)	26 (2)	83 (7)	35 (3)	139 (12)	5	353 (31)
10	1997–2014	776	238	48 (6)	46 (6)	29 (4)	12 (2)	29 (4)	8 (1)	62 (8)	1	319 (46)
11	2001–2004	501	8	38 (8)	10 (2)	21 (4)	15 (3)	49 (10)	19 (4)	48 (10)	1	120 (26)
12	2003–2014	1244	33	112 (9)	37 (3)	75 (6)	61 (5)	83 (7)	65 (5)	43 (4)	8	342 (32)
13	2007–2014	2587	50	83 (3)	176 (7)	2	60 (2)	212 (9)	49 (2)	170 (7)	1	1333 (59)
14	2006–2014	730	4	46 (3)	23 (3)	31 (4)	45 (6)	73 (11)	16 (2)	36 (5)	1	246 (41)
15	2011–2014	1107	6	35 (3)	41 (4)	13 (1)	87 (8)	117 (12)	36 (3)	65 (6)	6	519 (56)
16	2011–2012	701	9	54 (8)	17 (3)	0	20 (3)	70 (10)	20 (3)	59 (8)	0	365 (57)

Percentages are shown in brackets, where % ≥1.0. Endoscopies performed by doctors who carried out less than 500 are not shown unless they were the sole endoscopists over a period of one or more years (see Fig. 1). Findings differed by endoscopist for all disorders (*P* < 0.0001 for all, except for KS where *P* = 0.003). Variation in the length of time contributed reflects the contributions made by some volunteers on short term contracts, some recent trainees who now contribute to the service regularly, and two deaths among endoscopists in the 1990s. Trainees’ reports are always signed by the supervising doctor and are not reflected in this table until deemed ready for independent endoscopy

### Infectious and HIV-related disorders

**Fig. 2:**
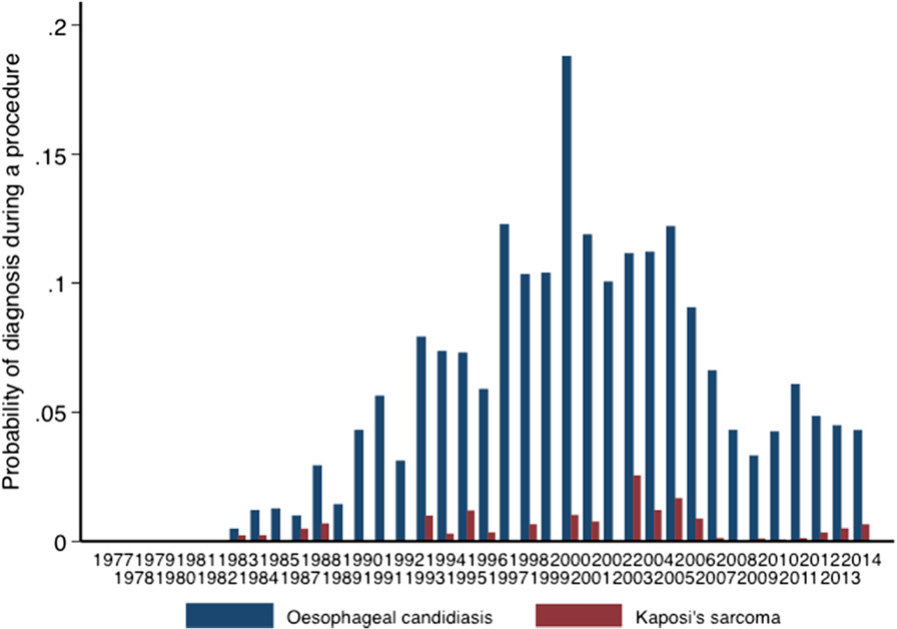
HIV-related disorders over time: oesophageal candidiasis and Kaposi’s sarcoma are shown. The first publication defining the emergence of AIDS in Zambia was published in 1984 [24], and antiretroviral treatment became available at scale through public health facilities in 2005

Two endoscopic diagnoses, oesophageal candidiasis and Kaposi’s sarcoma (KS), are AIDS-defining diagnoses. These diagnoses rose steeply during the early years of the HIV epidemic in Zambia and have declined since 2005, which is when antiretroviral therapy was scaled up across Zambia (Fig. 2).

### Peptic ulceration

**Fig. 3:**
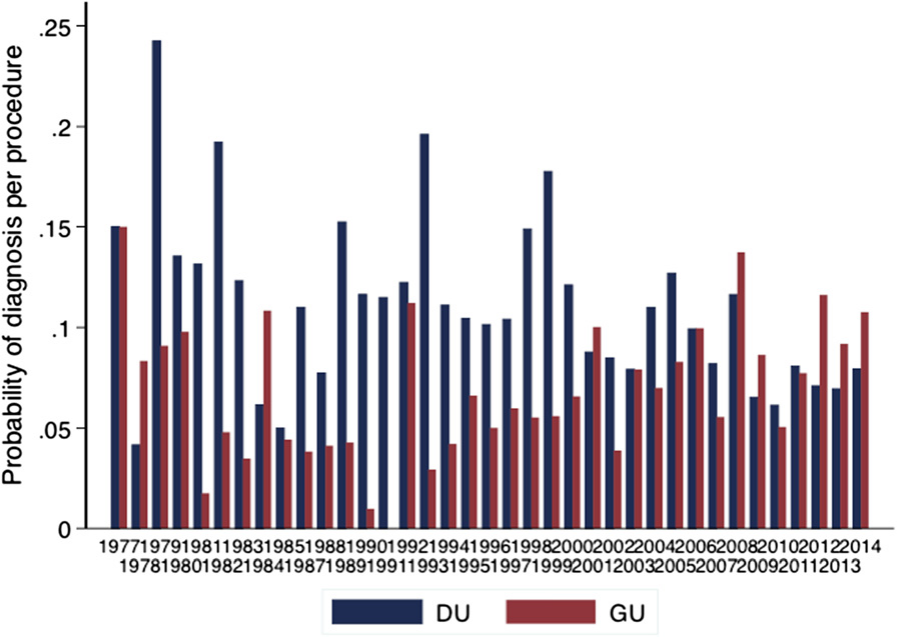
Gastric ulcer (GU) and duodenal ulcer (DU) diagnosis as percentage of peptic ulcer diagnoses over time

Gastric ulcer (GU) and duodenal ulcer (DU) have been diagnosed throughout this period, but differ in their secular trends, with GU rising over time and DU decreasing (Fig. 3). Using logistic regression to model the effects of time on DU in 15,467 records, there has been a 14 % reduction in DU diagnosis per decade (OR 0.86; 95 % CI 0.82, 0.90; *P* < 0.0001). Female sex was associated with a lower risk of DU (OR 0.64; 95 % CI 0.57, 0.71; *P* < 0.0001). In contrast, in 15,076 records there has been a steady rise in diagnosis of GU of 22 % per decade (OR 1.22; 95 % CI 1.15, 1.30; *P* < 0.0001), and again women were less likely to have a GU detected (OR 0.73; 95 % CI 0.64, 0.82; *P* < 0.0001). When surgical specialty or individual doctors were included in the regression models, these changes per decade remained highly significant, with little effect on odds ratio. In these models no individual doctor was specifically less or more likely to diagnose GU, but doctors 5 (a physician) and 12 (a surgeon) were significantly less likely to diagnose DU. DU, but not GU, was seasonal (*P* < 0.0001), with lower incidence (481, 8 %) in the hot season (October-December) than during the rainy or cool seasons (1023, 9.7 %).

### Upper gastrointestinal haemorrhage

We identified 1532 patients whose indication for endoscopy included haematemesis. The commonest endoscopic diagnosis was oesophageal varices, followed by DU and GU (Table 3). Within the group of patients presenting with haematemesis (*n* = 1517) there were trends in diagnosis. In logistic regression models, there was a 22 % increase per decade in GU (OR 1.22; 95 % CI 1.05, 1.41; *P* = 0.007) and a 14 % increase per decade in oesophageal varices (OR 1.14; 95 % CI 1.02, 1.28; *P* = 0.02), but no change in DU incidence (*P* = 0.42).

**Table 3:** Endoscopic findings in 1532 patients with haematemesis

	n (%)
Oesophageal varices	366 (24)
Duodenal ulcers	234 (15)
Gastric ulcers	216 (15)
Oesophagitis (reflux or unspecified)	54 (4)
Gastric carcinoma	43 (3)
Oesophageal ulcers	23 (1.5)
Mallory-Weiss tear	9 (0.6)
Oesophageal carcinoma	9 (0.6)
Kaposi’s sarcoma	2 (0.1)
No explanation found	576 (38)

### Presumptive diagnosis of upper gastrointestinal malignancy

Oesophageal cancer was presumptively diagnosed in 549 (3.3 %) procedures. This cancer was reported more frequently in recent years, especially so in younger patients (Table 4). In logistic regression models, we estimate that there is a 32 % increase in diagnosis of oesophageal cancer per decade (OR 1.32; 95 % CI 1.20, 1.45; *P* < 0.0001), and this rises to 66 % increase per decade in patients under 45 years of age (OR 1.66; 95 % CI 1.30, 2.10; *P* < 0.0001). The frequency of diagnosis of oesophageal cancer in patients over 60 remained unchanged. The logistic regression models suggest that women are less likely to be diagnosed with cancer (OR 0.56, 95 % CI 0.47,0.68; *P* < 0.0001); this effect was not observed in patients under 45 years of age (*P* = 0.29). The temporal trends in oesophageal cancer remained apparent and even increased after adjusting for doctor such that the rise in diagnosis by decade increased to 53 %. In these models, doctors, 7 (surgeon), 9 (physician), 10 (paediatrician) and 13 (physician) made fewer diagnoses of oesophageal cancer.

**Table 4:** Age distribution of upper GI malignancy

Age group (years)	1977–1985^a^	1987–1996	1997–2006	2007–2014	Total
Oesophageal cancer					
16–44	4 (13)	13 (31)	12 (21)	82 (27)	111 (25)
45–59	14 (47)	17 (40)	22 (39)	102 (33)	155 (35)
60 and over	12 (40)	12 (29)	22 (39)	125 (40)	171 (39)
Gastric cancer					
16–44	4 (19)	12 (29)	38 (37)	46 (22)	100 (27)
45–59	8 (38)	13 (32)	25 (24)	67 (33)	113 (31)
60 and over	9 (43)	16 (39)	39 (38)	92 (45)	156 (42)

^a^No data from 1986 retrieved. Decades are not uniform as in Table 1

Gastric cancer overall did not show this temporal trend (OR 1.10; 95 % CI 0.98, 1.21; *P* = 0.056), but in patients under 60 years of age there was a trend, with increasing detection rates per decade (OR 1.21; 95 % CI 1.03, 1.43; *P* = 0.02). This trend was not apparent in patients under 45 years of age. In patients of 60 years of age or more the frequency of diagnosis of gastric cancer appears to have declined by 15 % per decade (*P* = 0.03).

### Endoscopic findings in children

The records include 527 children (3.5 % of 14,944 where the age can be ascertained), where children are defined as 16 years or below. Only five doctors carried out endoscopy on children, one a paediatrician (238 procedures of all and 39/70 (55 %) of those in children under 5 years of age). The majority were normal, but peptic ulceration and oesophageal varices were common also (Table 5).

**Table 5:** Endoscopic findings in children

Rank order	Oesophagus, *n* = 510	Stomach, *n* = 501	Duodenum, *n* = 462
1	Normal 400 (78)	Normal 417 (83)	Normal 385 (83)
2	Varices 49 (10)	Gastritis 44 (9)	Duodenal ulcers 41 (9)
3	Stricture 16 (3)	Gastric ulcers 17 (3)	Duodenitis 26 (6)
4	Oesophagitis 7 (3)	Gastric outlet obstruction 9 (2)	Vascular lesion 4 (1)
5	Hiatus hernia 7 (1)	Growths/polyps 5 (1)	Villous atrophy 3 (1)
6	Ulcers 4 (1)	Gastric erosions 4 (1)	Polyp 2 (0.5)
7	Other 1 (0.2)	Vascular lesions 2 (0.5)	Stricture 1 (0.2)

Endoscopic findings in children in descending order of frequency n (%). Percentages are shown in brackets

### Other non-communicable disorders

Reflux oesophagitis was recorded in 399 (2.4 %) procedures, though with some inter-observer variation (see above). Hiatus hernia was recorded in 110 (0.7 %) and non-specific oesophagitis (presumably often reflux) in 532 (3.2 %). However, Barrett’s oesophagus was rare, with only 10 cases recorded. Stricture was found in 540 (3.2 %) patients. Gastric outlet obstruction was found in 314 (1.9 %). Over the last few years we have become increasingly aware that caustic ingestion (frequently parasuicide) is a common cause of oesophageal strictures and gastric outlet obstruction. These findings were infrequently recognised as due to caustic injury and often appear as unexplained. In 114 cases where caustic ingestion was recorded as the indication for endoscopy, oesophageal stricture was found in 41 (36 %) and gastric outlet obstruction in 36 (32 %).

### Major indications for endoscopy

Abdominal or epigastric pain was the commonest indication for endoscopy, with 9798 recorded with this indication. Of 9323 procedures which were complete (ie no obstruction to the passage of the gastroscope, 4483 (48 %) were normal. If the incomplete procedures are assumed to have been abnormal, then 46 % of all procedures for abdominal pain were normal. Other prominent findings in patients with abdominal pain were DU (1005, 10 %), GU (664, 6.8 %), and gastric cancer (205, 2.1 %).

**Fig. 4:**
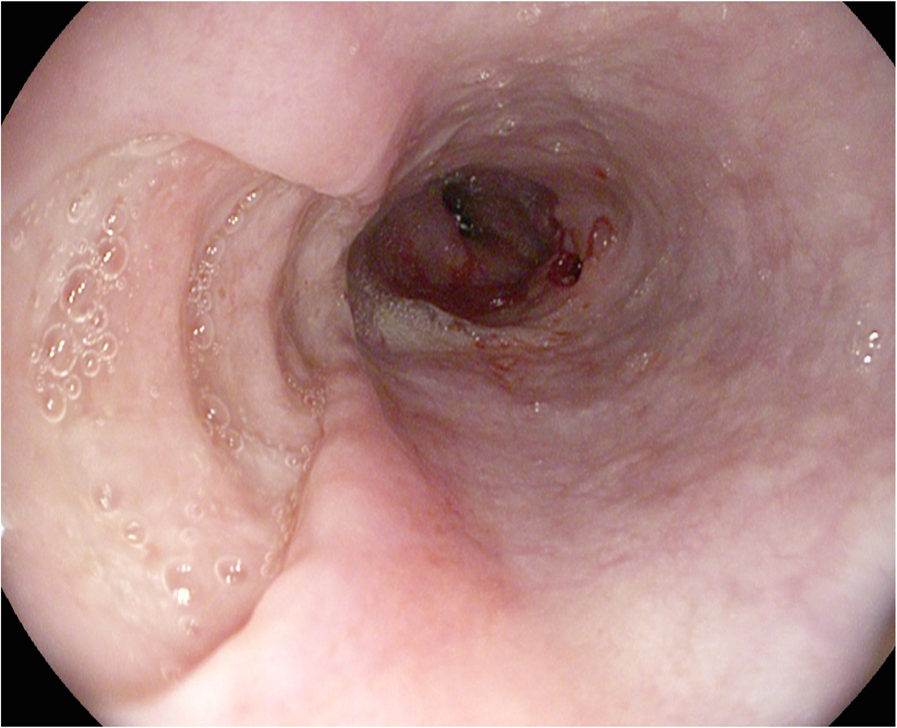
Giant oesophageal ulcers of HIV

Dysphagia was the indication for endoscopy in 1341 (8.6 %) of 15,632 patients whose indication could be ascertained. Of 1308 whose oesophagus was completely examined, 354 (27 %) had benign strictures and 360 (27.5 %) had tumours; 346 (26.5 %) were normal; 44 (3.4 %) had oesophageal ulcers, many of whom would have had giant oesophageal ulcers of HIV (Fig. 4); and 28 (2.1 %) had achalasia.

Anaemia was the indication for endoscopy in 294 (1.9 %) of 15,632 procedures. Findings in patients referred for anaemia included GU in 28 (9.7 %), DU in 24 (8.2 %), gastric cancer in 13 (4.4 %), oesophageal varices in 21 (7.5 %) and oesophageal cancer in 2 (0.7 %). Anaemia as an indication for endoscopy was associated with a borderline increased probability of diagnosis of gastric cancer (OR 1.73, 95 % CI 1.01, 2.96; *P* = 0.06) and oesophageal varices (OR 1.59, 95 % CI 1.04, 2.42; *P* < 0.05), but not peptic ulceration, which was no more frequent than in the whole dataset.

## Discussion

The substantial time span of this diagnostic record affords an interesting insight into the profile of upper gastrointestinal disease in Zambia over the last 4 decades, with evidence of important trends. Retrospective analysis of data like these is inevitably fraught with difficulties of interpretation, notably the inevitable turnover of staff which when combined with inter-observer variation generates changes in diagnostic patterns. However, the length of service of a few doctors who contributed the most to the record provides some consistency in diagnostic language, which may ameliorate some of these uncertainties. Furthermore, doctor 8 (Fig. 1) also taught endoscopy to doctors 9, 11, 12, 13, 14 and 15, which reinforces a consistency in the diagnostic nosology and helps to reduce inter-observer variation. There are differential trends for peptic ulceration (GU increasing and DU decreasing, and increasing diagnosis of oesophageal but not gastric cancer) so these changes are not merely a consequence of greater use of the endoscopy unit.

The burden imposed by HIV on the population of Zambia over the period of this analysis has been heavy, and this is reflected in the dramatic changes in oesophageal candidiasis and Kaposi’s sarcoma. The first description of the new ‘epidemic’ form of KS in this same hospital was published in 1984 [24] and it is clear from Fig. 2 that gastrointestinal KS and oesophageal candidiasis were both rare before then, even though HIV must have been circulating. The steep rise in these diagnoses in the 1980s and 1990s reflects the rising HIV seroprevalence over those years. It is satisfying to note the substantial fall in both diagnoses since the widespread use of ART. These findings are consistent with findings in Zimbabwe and Uganda where a reduction in KS cases (not gastrointestinal) has been reported during the ART era [25, 26]. We have also shown that oesophageal candidiasis, once the most common finding in patients with HIV infection, has been declining with more widespread use of ART. This was observed in the USA also [27]. In addition to reduced incidence of candidiasis we have also seen a reduction in severity which is hard to measure; in the 1990s candidiasis was sometimes so severe as to block the air/water channel of the endoscope, but this is very rare now. Cases are still seen, however, and anecdotally we find that most of these occur in patients who have not had a diagnosis of HIV.

The burden of cancer is increasing in Sub-Saharan Africa with the burden expected to double within the next 20 years [28] leading to an estimated 1 million cancer deaths per year by 2030 [29]. The temporal trends in endoscopic diagnosis of upper GI malignancy we report here are of particular concern. Both oesophageal cancer and gastric cancer appear to have risen in adults under 60 years of age. In contrast, in patients of 60 years of age or more the frequency of diagnosis of gastric cancer appears to have declined by 15 % per decade, and the frequency of diagnosis of oesophageal cancer in patients over 60 has remained unchanged. These differential trends suggest that the changes are not merely due to improved endoscopic equipment or changes in personnel, but we cannot rule out the possibility that these temporal trends may be influenced by changes in awareness and referral pattern. As this was a retrospective review, we could not confirm the histological diagnosis in these cancer cases. However, GI cancers in Africa often present with advanced, unmistakeable lesions, and the high proportion of cancer in young adults is consistent with our findings in recent studies in which the diagnosis was confirmed histologically [30–32]. In the UK, less than 5 % of upper GI cancers are under 50 years of age [33], but a report from Tanzania 29 % of gastric cancer cases were under 50 years of age [34], and this is true of oesophageal cancer in Ghana [35] and Kenya [36]. Our findings may therefore be representative of Africa more widely.

An important contributor to the temporal trends is referral pattern. When the endoscopy service first began, in 1977, health care in Zambia was free at the point of care and was fully supported by the government. After 1991 when most of the state’s assets were being privatised and the economy could no longer support free health care for all, cost sharing user fees were introduced in government hospitals including UTH. A state insurance scheme was later introduced but suffered from the drawback that, as it was not compulsory, contributions were often only made when the potential contributor was feeling ill and wanted care, so it was in effect a form of user fee. Currently, medical care remains free for children and elderly patients and for those with communicable diseases such as HIV infection or Tuberculosis. Diagnostic services attract fees, and the fee for endoscopy is now ZMW 150 (USD 22 approximately), which represents a considerable barrier to investigation as 60.5 % of the population in Zambia are living in poverty and can barely afford it. User fees have thus been applied to endoscopy for almost all of the last 24 years. While these changes have been going on, referral patterns have almost certainly changed, but in a way that is difficult to quantify. Awareness of the endoscopy service around the country has certainly changed over the last four decades, and this may help explain the rising diagnostic rate of oesophageal varices. Our analysis may suffer from a selection bias as only those who could afford user fees and transport costs would have been able to attend the endoscopy unit. The cohort selection could also have been affected by referral awareness by the referring doctors and easy of geographical accessibility to the service. In order to reduce these biases, we have in our analyses considered proportions of diagnoses made as opposed to absolute numbers.

The proportion of patients who were referred for abdominal pain who had a normal endoscopy was high at 48 %. This figure is consistent with reports from other countries and is consistent with our clinical impression that functional dyspepsia is just as common in Africa as elsewhere. Oesophageal varices were found to be the most common cause of haematemesis, and most of these are attributed to *Schistosoma mansoni* infection [30, 37]. Other African investigators within the region have also reported oesophageal varices as the leading cause of haematemesis, in contrast to the dominance of peptic ulceration in industrialised countries [30, 38, 39], but this may vary across the continent. Duodenal ulcer (DU) is assumed to be due to *H. pylori* infection, which is common [18], but this is not routinely confirmed as confirmation is costly and probably unnecessary in the light of the high seroprevalence. The seasonality of DU, with lower incidence in the hot season, is consistent with data from other countries [40] but remains unexplained.

These data from one endoscopy unit provide a useful insight into profiles of upper gastrointestinal disease in Zambia, and they differ substantially from current trends in industrialised countries. There is a serious shortage of data on the epidemiology of gastrointestinal disease in Africa [41], but we can predict that further changes will occur, and these may be rapid in the increasing number of people surviving long term with HIV infection [42]. These endoscopy records go some way to identifying issues which require urgent investigation, prominent among which is the disturbing incidence of oesophageal and gastric cancer in young adults.

## Conclusions

These data from an African endoscopy unit suggest that the profile of upper gastrointestinal disease is evolving over time. The profile of diagnosis has some important local characteristics, such as the dominance of oesophageal varices as a cause of haemorrhage which other evidence suggests is largely due to schistosomiasis. There are also common features which would be found in any endoscopy unit anywhere in the world. The emerging problem of cancer in younger adults, and the apparent changes in peptic ulceration, merit further study.

## References

[1] Sonnenberg A (2013). Historic changes of Helicobacter pylori-associated diseases. Aliment Pharmacol Ther.

[2] Goldacre MJ (2009). Demography of aging and the epidemiology of gastrointestinal disorders in the elderly. Best Pract Res Clin Gastroenterol.

[3] Rustgi AK, El-Serag HB (2014). Esophageal cancer. NEJM.

[4] Loffeld RJ, Liberov B, Dekkers PE (2012). The changing prevalence of upper gastrointestinal endoscopic diagnoses: a single-centre study. Neth J Med.

[5] National Cancer Intelligence Network. Incidence of stomach cancer in England, 1998–2007. http://www.ncin.org.uk/publications/data_briefings/incidence_of_stomach_cancer_in_England, accessed on 13th February 2015.

[6] Vyse AJ, Gay NJ, Hesketh LM, Andrews NJ, Marshall B, Thomas HI, et al. The burden of Helicobacter pylori infection in England and Wales. Epidemiol Infect 2002;128:411-7. 10.1017/s0950268802006970 PMC2869837 12113485

[7] Pennathur A, Gibson MK, Jobe BA, Luketich JD (2013). Oesophageal carcinoma. Lancet.

[8] Devesa SS, Blot WJ, Fraumeni JF (1998). Changing patterns in the incidence of esophageal and gastric carcinoma in the United States. Cancer.

[9] Kocher HM, Linklater K, Patel S, Ellul JP (2001). Epidemiological study of oesophageal and gastric cancer in south-east England. Br J Surg.

[10] Xia B, Xia HH, Ma CW, Wong KW, Fung FM, Hui CK, et al. Trends in the prevalence of peptic ulcer disease and Helicobacter pylori infection in family physician-referred uninvestigated dyspeptic patients in Hong Kong. Aliment Pharmacol Ther 2005; 22: 243-9. 10.1111/j.1365-2036.2005.02554.x 16091062

[11] Wong SN, Sollano JD, Chan MM, Carpio RE, Tady CS, Ismael AE, et al. Changing trends in peptic ulcer prevalence in a tertiary care setting in the Philippines: a seven-year study. J Gastroenterol Hepatol. 2005;20:628-32. 10.1111/j.1440-1746.2005.03719.x 15836714

[12] Nakajima S, Nishiyama Y, Yamaoka M, Yasuoka T, Cho E (2010). Changes in the prevalence of Helicobacter pylori infection and gastrointestinal diseases in the past 17 years. J Gastroenterol Hepatol.

[13] Goh KL (2007). Changing trends in gastrointestinal disease in the Asia-Pacific region. J Dig Dis.

[14] Saul C, Teixeira CR, Pereira-Lima JC, Torresini RJ (2007). Prevalence reduction of duodenal ulcer: a Brazilian study (retrospective analysis in the last decade: 1996–2005). Arq Gastroenterol.

[15] Somdyala NI, Parkin DM, Sithole N, Bradshaw D (2015). Trends in cancer incidence in rural Eastern Cape Province; South Africa, 1998–2012. Int J Cancer.

[16] Holcombe C (1992). Helicobacter pylori: the African enigma. Gut.

[17] Tanih NF, Dube C, Green E, Mkwetshana N, Clarke AM, Ndip LM (2009). An African perspective on Helicobacter pylori: prevalence of human infection, drug resistance, and alternative approaches to treatment. Ann Trop Med Parasitol.

[18] Fernando N, Holton J, Zulu I, Vaira D, Mwaba P, Kelly P (2001). Helicobacter pylori infection in an urban African population. J Clin Microbiol.

[19] http://www.worldgastroenterology.org/assets/downloads/en/pdf/guidelines/11_helicobacter_pylori_developing_countries_en.pdf, Accessed on 21/01/15.

[20] Parkin DM, Bray F, Ferlay J, Jemal A (2014). Cancer in Africa 2012. Cancer Epidemiol Biomarkers Prev.

[21] Ferlay J, Soerjomataram I, Ervik M, Forman D, Bray F, Dikshit R, et al. GLOBOCAN 2012 v1.0, Cancer Incidence and Mortality Worldwide: IARC CancerBase No. 11 [Internet]. Lyon, France: International Agency for Research on Cancer; 2013. Available from: http://globocan.iarc.fr, accessed 18/10/2014.

[22] Mbulaiteye SM, Bhatia K, Adebamowo C, Sasco AJ (2011). HIV and cancer in Africa: mutual collaboration between HIV and cancer programs may provide timely research and public health data. Infect Agent Cancer.

[23] Dominguez RL, Crockett SD, Lund JL, Suazo LP, Heidt P, Martin C, et al. Gastric cancer incidence estimation in a resource-limited nation: use of endoscopy registry methodology. Cancer Causes Control 2013;24:233-9. 10.1007/s10552-012-0109-5 PMC3815449 23263776

[24] Bayley AC (1984). Aggressive Kaposi’s sarcoma in Zambia, 1983. Lancet.

[25] Chokunonga E, Borok MZ, Chirenje ZM, Nyakabau AM, Parkin DM (2013). Trends in the incidence of cancer in the black population of Harare, Zimbabwe 1991–2010. Int J Cancer.

[26] Wabinga HR, Nambooze S, Amulen PM, Okello C, Mbus L, Parkin DM (2014). Trends in the incidence of cancer in Kampala, Uganda 1991–2010. Int J Cancer.

[27] Monkemuller KE, Call SA, Lazenby AJ, Wilcox CM (2000). Declining prevalence of opportunistic gastrointestinal disease in the era of combination antiretroviral therapy. Am J Gastroenterol.

[28] Jemal A, Bray F, Forman D, O'Brien M, Ferlay J, Center M, et al. Cancer burden in Africa and opportunities for prevention. Cancer 2012;118:4372-84. 10.1002/cncr.27410 22252462

[29] Sylla BS, Wild CP (2012). A million Africans a year dying from cancer by 2030: what can cancer research and control offer to the continent?. Int J Cancer.

[30] Kelly P, Katema M, Amadi B, Zimba L, Aparicio S, Mudenda V, et al. Gastrointestinal pathology in the University Teaching Hospital, Lusaka, Zambia - review of endoscopic and pathology records. Trans Roy Soc Trop Med Hyg 2008;102:194-199. 10.1016/j.trstmh.2007.10.006 18054058

[31] Kayamba V, Asombang AW, Mudenda V, Lisulo MM, Sinkala E, Mwanamakondo S (2013). Gastric adenocarcinoma in Zambia: a case–control study of HIV, lifestyle risk factors, and biomarkers of pathogenesis. S Afr Med J.

[32] Kayamba V, Bateman AC, Asombang A, Shibemba A, Zyambo K, Banda T, et al. HIV infection and domestic smoke exposure, but not human papillomavirus, are risk factors for oesophageal squamous cell carcinoma in Zambia: a case-control study, Cancer Medicine, 2014 in press. doi: 10.1002/cam4.434. 10.1002/cam4.434 PMC4402073 25641622

[33] Coupland VH, Allum W, Blazeby JM, Mendall MA, Hardwick RH, Linklater KM, et al. Incidence and survival of oesophageal and gastric cancer in England between 1998 and 2007, a population-based study. BMC Cancer 2012;12:11. 10.1186/1471-2407-12-11 PMC3274437 22239958

[34] Mabula JB, McHembe MD, Koy M, Chalya PL, Massaga F, Rambau PF, et al. Gastric cancer at a university teaching hospital in northwestern Tanzania: a retrospective review of 232 cases. World J Surg Oncol 2012;10:257. 10.1186/1477-7819-10-257 PMC3527214 23181624

[35] Tettey M, Edwin F, Aniteye E, Sereboe L, Tamatey M, Ofosu-Appiah E, et al. The changing epidemiology of esophageal cancer in sub-Saharan Africa - the case of Ghana. Pan Afr Med J. 2012;13:6. PMC3527059 23308313

[36] Parker RK, Dawsey SM, Abnet CC, White RE (2010). Frequent occurrence of esophageal cancer in young people in western Kenya. Dis Esophagus.

[37] Sinkala E, Kapulu M, Besa E, Zyambo K, Chisoso NJ, Foster GR, et al. Hepatosplenic schistosomiasis is characterised by high blood markers of translocation, inflammation and fibrosis. Liver Int. In press. 10.1111/liv.12891 26058680

[38] Alema ON, Martin DO, Okello TR (2012). Endoscopic findings in upper gastrointestinal bleeding patients at Lacor hospital, northern Uganda. Afr Health Sci.

[39] Jaka H, Koy M, Liwa A, Kabangila R, Mirambo M, Scheppach W, et al. A fibreoptic endoscopic study of upper gastrointestinal bleeding at 10.1186/s12876-015-0353-8 Bugando Medical Centre in northwestern Tanzania: a retrospective review of 240 cases. BMC Res Notes 2012;5:200. 10.1186/1756-0500-5-200 PMC3392734 22537571

[40] Fares A (2013). Global patterns of seasonal variation in gastrointestinal diseases. J Postgrad Med.

[41] Asombang AW, Kelly P (2012). Gastric cancer in Africa: what do we know about incidence and risk factors?. Trans R Soc Trop Med Hyg.

[42] Kelly P, Saloojee H, Chen J, Chung RT (2014). Non-communicable disease in HIV infected adults in Africa: gastrointestinal, hepatic and nutritional disorders. JAIDS.

